# Molecular investigation of *Torque teno sus virus* in geographically distinct porcine breeding herds of Sichuan, China

**DOI:** 10.1186/1743-422X-10-161

**Published:** 2013-05-24

**Authors:** Miao Mei, Ling Zhu, Zhiwen Xu, Ling Zhao, Yuancheng Zhou, Yunfei Wu, Song Li, Haoche Wei, Wanzhu Guo

**Affiliations:** 1 Animal Biotechnology Center, College of Veterinary Medicine, Sichuan Agricultural University, Ya’an 625014, China; 2 Key Laboratory of Animal Disease and Human Health in Sichuan Province, College of Veterinary Medicine, Sichuan Agricultural University, Ya’an 625014, China; 3 Beijing Centre Biology Co. Ltd, Beijing 102206, China

**Keywords:** *Torque teno sus virus* (TTSuV), Porcine, Prevalence, Genogroup, Subtype, Phylogenetic analysis

## Abstract

**Background:**

*Torque teno sus virus* (TTSuV), infecting domestic swine and wild boar, is a non-enveloped virus with a circular, single-stranded DNA genome. which has been classified into the genera *Iotatorquevirus* (TTSuV1) and *Kappatorquevirus* (TTSuV2) of the family *Anelloviridae*. A molecular study was conducted to detect evidence of a phylogenic relationship between these two porcine TTSuV genogroups from the sera of 244 infected pigs located in 21 subordinate prefectures and/or cities of Sichuan.

**Results:**

Both genogroups of TTSuV were detected in pig sera collected from all 21 regions examined. Of the 244 samples, virus from either genogroup was detected in 203 (83.2%), while 44 animals (18.0%) were co-infected with viruses of both genogroups. Moreover, TTSuV2 (186/244, 76.2%) was more prevalent than TTSuV1 (61/244, 25%). There was statistically significant difference between the prevalence of genogroups 1 infection alone (9.4%, 23/244) and 2 alone (64.8%, 158/244), and between the prevalence of genogroups 2 (76.2%, 186/244) and both genogroups co-infection (18.0%, 44/244). The untranslated region of the swine TTSuV genome was found to be an adequate molecular marker of the virus for detection and surveillance. Phylogenetic analysis indicated that both genogroups 1 and 2 could be further divided into two subtypes, subtype a and b. TTSuV1 subtype b and the two TTSuV2 subtypes are more prevalent in Sichuan Province.

**Conclusions:**

Our study presents detailed geographical evidence of TTSuV infection in China.

## Background

*Torque teno sus virus* (TTSuV) is a member of the *Anelloviridae* family. There are currently two known species of TTSuV, TTSuV1 (genus *Iotatorquevirus*) and TTSuV2 (genus *Kappatorquevirus*) [1,2]. TTSuV is a small icosahedral, non-enveloped virus with a single-stranded (ss), negative sense, circular DNA genome which can infect domestic swine and wild boar [2]. Members of the *Anelloviridae* family can infect many vertebrate animals including humans, domestic swine, wild boars, chicken, sheep, cattle, dogs, cats and non-human primates [3,4]. In humans, these viruses are ubiquitous and several genogroups have been identified [5]. Analysis of viral genomic DNA has revealed well-conserved genomic organization among the various *Anelloviridae* family members [6,7]. However, *Anelloviruses* of different species showed distinct genome lengths and large variability among their sequences. The genome size of species-specific *Anelloviruses* vary from 2.1 to 3.8 kb. The genomes of TTSuVs that infect pigs are approximately 2.8 kb [4]. Porcine TTSuVs contain 3 or 4 partially overlapping open reading frames, and a short untranslated region (UTR) with high GC content [8,9]. The characteristics of TTSuV nucleotide and amino acid motifs were reported previously [8]. Four prototype USA strains of TTSuV were isolated from a single pig [8]. These strains showed distinct genotypes or subtypes, therefore a revised classification system for TTSuV was proposed. Nested polymerase chain reaction (PCR), based on the conserved regions in the UTR of TTSuV1 and TTSuV2, has been widely employed to detect these viruses [10-12].

To date, there is no confirmed case associating Torque teno virus (TTV) infection with a specific disease in humans [13-15]. The prevalence of human TTV infection differs widely depending on age and geographical location [16,17]. Detection of viral nucleic acid in cord blood, semen, the serum of mother-to-child pairs, breast milk, saliva, nasal secretions and feces suggests that TTV can be transmitted vertically and horizontally [5,14,16,18-20].

Porcine TTSuVs have been detected by PCR in pig sera of many different countries, including the USA, Canada, Korea, China, Thailand, France, Italy, Spain, and Uganda, with frequencies ranging from 16.8–100%, but detection of these viruses was not associated with populations, sanitary situation or biosecurity [6,10,21-25]. In an epidemiology study of TTSuV in central China, 15% tissue samples from post-weaning multisystemic wasting syndrome (PMWS)-affected pigs and 75% blood samples from healthy pigs were found positive for TTSuV1 and/or 2. Phylogenetic analysis based on complete genomes suggested that the causative agents were TTSuV subtypes 1b and 2d [26]. Moreover, the genetic diversity of these viruses were found based on 5′ non-coding genes in swine herds experiencing clinical symptoms of 11 different regions of China (Anhui, Beijing, Guangxi, Henan, Hunan, Jiangsu, Jiangxi, Shan-dong, Shanghai, Xinjiang and Zhejiang) during 2008–2009. Their results revealed a high TTSuV-positive rate of 78.9%, and concurrent infections with multiple TTSuV strains in the same pig [27].

Several recent studies have reported that TTSuV viral loads and biological characterstic differ between healthy and diseased porcine herds determined by quantitative PCR (qPCR) [28-31]. In addition to serum, both TTSuV genogroups have been detected in plasma, semen, feces, colostrum and stillborns samples [18,32,33]. It is believed that TTSuV infection is both horizontal and vertical [33,34]. TTSuV infection appears early during production and spread in the farrowing crates [35,36]. Differences observed in different genogroups of TTSuV infection could be due to the pig breed [37]. TTSuV has been found in sera, plasma, feces, veterinary vaccines, cell cultures, trypsin and samples that are commonly used as laboratory reagents. These results indicated that fecal-oral transmission and injection of vaccines were the most likely routes of transmission [32,38,39]. Pig herds at high densities will likely facilitate TTSuV infection [11]. Previous studies reported that TTSuV was also detected in brain, lung, heart, liver, spleen, kidney, bone marrow, mediastinal and mesenteric lymph nodes at different ages [40]. Dynamics of infection and excretion of TTSuVs throughout the productive life of animals were also described [36]. Additionally, high prevalence of TTSuVs was observed in dozens of species of European wild boars (*Sus scrofa*) [9,11]. The virulence of TTSuV genogroups to induce specific diseases remain unknown [31,41,42]. TTSuV1 could be a potential viral pathogen for the domestic pig, as infection with TTSuV1 is associated with characteristic pathological changes in gnotobiotic pigs [41]. Our research group also found that infection with TTSuV2 alone was able to induce certain pathogenic lesions in specific pathogen-free piglets [42]. A relationship between TTSuV infection and PMWS, a disease caused by porcine circovirus type 2 (PCV2) infection, has been shown. High viral loads of of TTSuV2 infection has been associated with PMWS-affected pigs as shown by qPCR [10]. Although TTSuV1 infecion prior to PCV2 infection in gnotobiotic piglets facilitated the development of PMWS [43], TTSuV1 prevalence and load were not related to PCV diseases [44]. Furthermore, co-infection of porcine TTSuV1 with porcine reproductive and respiratory syndrome virus (PRRSV) has been linked with the development of a porcine dermatitis and nephropathy syndrome-like condition in gnotobiotic pigs [45]. Natural infection with TTSuV1 suppressed the immune response to PRRSV vaccination [46].

Little information regarding the epidemiology of TTSuV genogroups is available. This study investigated the prevalence and genetic diversity as well as epidemiology of TTSuV1 and TTSuV2 using species-specific UTR nested PCR methods in healthy animals of Sichuan province, China.

## Results

### TTSuV prevalence among regional breeds of pigs

**Figure 1:**
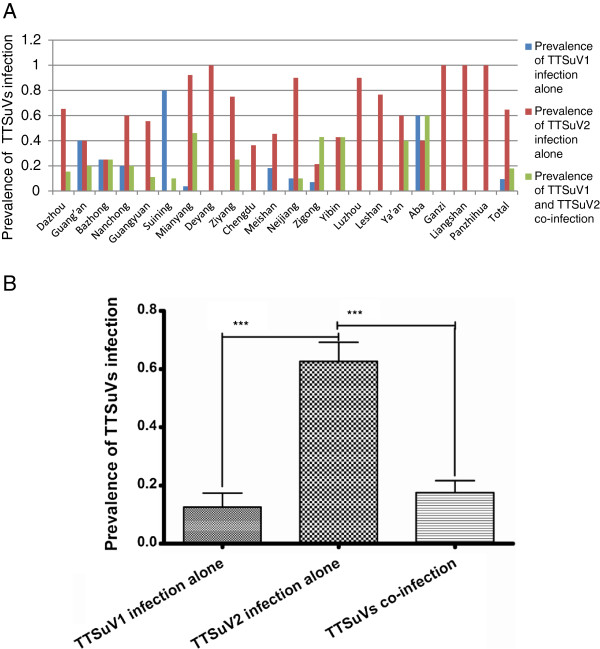
**Prevalence of torque teno sus virus infection in different breeding herds of Sichuan, China.** Prevalence of torque teno sus virus infection in geographically distinct breeding herds of different cities of Sichuan, China. Total prevalence of torque teno sus virus genogroups infection in Sichuan, China (***p < 0.001).

Using nested-PCR technique to amplify target fragments, we found that 83.2% (203/244) of domestic swine tested were positive for one of the TTSuV genogroups. There was difference in the prevalence of TTSuVs infection in geographically distinct porcine herds. TTSuV1 infection was not found in seven of 21 prefectures and cities (Figure 1A). There was statistically significant difference between the prevalence of genogroups 1 infection alone (9.4%, 23/244) and 2 alone (64.8%, 158/244), and between the prevalence of genogroups 2 (76.2%, 186/244) and both genogroups co-infection (18.0%, 44/244) (Figure 1B). Moreover, TTSuV2 (186/244, 76.2%) was more prevalent than TTSuV1 (61/244, 25%).

### TTSuV UTR sequences as a molecular marker

**Figure 2:**
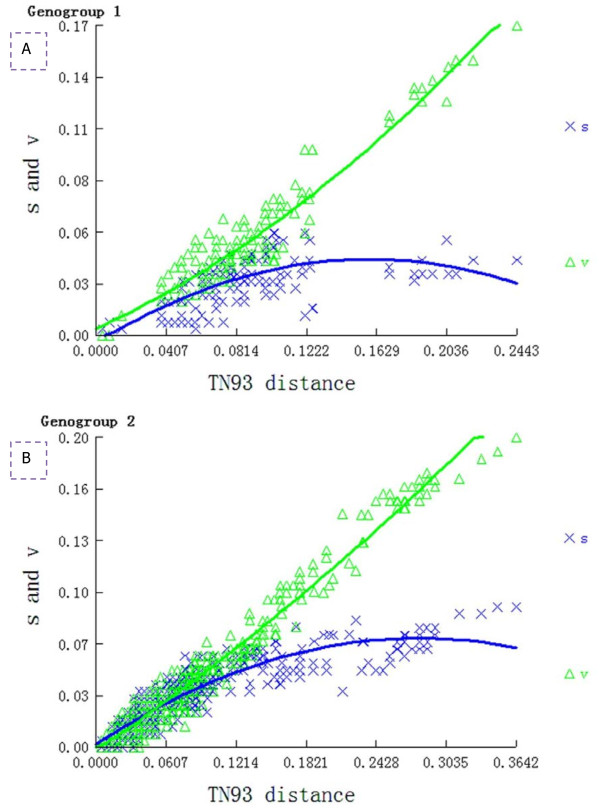
**The number of transitions (×) and transversions (∆) versus of the genetic distance calculated with the TN93 among all pairwised strains of TTSuV1 (A) and 2 (B).** Solid lines indicate the best fit found in each mutational type. The “s”and “v” represented the number of transitions (**×**) and transversions (∆), respectively.

The absolute number of transitions and transversions versus genetic distance were plotted (Figure 2). The number of observed transversions relative to transitions gradually increased with increasing divergence. Both data sets resembled a line, revealing that transversions and transitions were not saturated. Moreover Xia’s test supported little saturation for TTSuV genogroups 1 and 2 (Iss < Iss.c, *p* < 0.0005). A neutral model of selection for the DNA section tested was supported by the non-significant results of Tajima’s D, and Fu and Li’s test, for the entire dataset, and for TTSuV genogroups 1 and 2 separately. The levels of nucleotide molecular diversity per site between two sequences (0.08242 *vs.* 0.09334) and average number of pairwise nucleotide discrepancies (10.137 *vs.* 10.360) were slightly lower in TTSuV1. Mean distances within TTSuVs (0.0792 ± 0.0131 *vs.* 0.1387 ± 0.0134) were calculated using the p-distance of MEGA5. DNA substitution models for all available data were HKY + G [Akaike Information Criterion (AIC) weight all = 0.3452], F81 + G (AIC weight1 = 0.1424) and HKY + G (AIC weight2 = 0.5114).

The estimated transition/transversion bias (R) between the complete dataset and both genogroups, TTSuV1 and TTSuV2 was 0.75, 0.37 and 0.68, respectively. Substitution patterns and rates were estimated using the Tamura-Nei (1993) model (TN93). A discrete Gamma distribution was used to model evolutionary rate differences among sites (5 categories, parameters were equal to 2.7105, 0.8833 and 0.5607) with assistance from MEGA5. The nucleotide frequencies for all datasets were different, (both genogroups: A = 38.51%, T/U = 26.13%, C = 16.25%, and G = 19.11%; genogroup 1: A = 30.62%, T/U = 28.82%, C = 16.67%, and G = 23.89%; genogroup 2: A = 41.15%, T/U = 24.52%, C = 16.68%, and G = 17.64%). For estimating the Maximum Likelihood (ML) values, a user-specified toplogy was used. The maximum Log likelihood for this computation was− 1347.065, -522.686 and −813.832, for both genogroups, genogroup 1 and genogroup 2, respectively. This analysis involved 18 TTSuV1 and 37 TTSuV2 nucleotide sequences. All positions with less than 95% site coverage were eliminated. There were a total of 123 positions for TTSuV1 and 112 positions for TTSuV2 in the final dataset.

### Sequencing and phylogenetic analysis of TTSuV UTR sequences

Analysis of the obtained sequences revealed a low genetic diversity within TTSuV genogroup variants but high genetic diversity between genogroups. Alignments contained a 225 bp overlapping sequence for genogroup 1, and a 184 bp overlapping sequence genogroup 2; these were used to calculate phylogenetic distances. Pairwise comparison of nucleotide sequences within a genogroup showed a high level of homology: 82.3–100% within TTSuV 1, and 72.2–100% within TTSuV2. The sequence identity among different variants was not apparently associated with geographical regions. Diversity between genogroups 1 and 2 sequences containing around 125 overlapping sequences not including primer motifs was high, with overall sequence identities of 27.6–67.5%.

**Figure 3:**
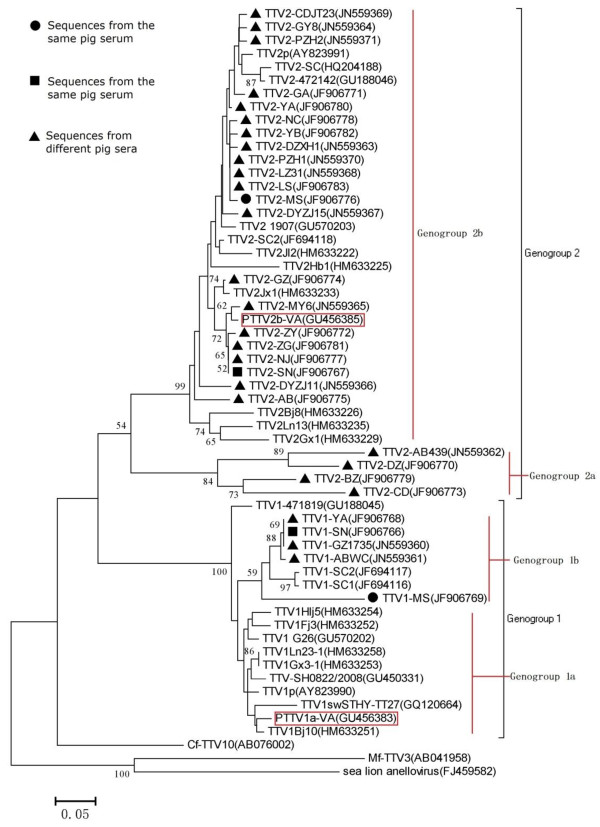
**Phylogenetic tree generated on a 125 bp overlapping stretch of the UTR of TTSuV isolates in this study and 28 reference strains.** Three other *Anellovirus* from different species were used as outgroups in this study. The neighbor-joining (NJ) method was used to construct this tree, which was evaluated using the interior branch test (Mega 5.0). Only bootstrap support values higher than 50% are shown at each node. ML (Maximum Likelihood), MP (Maximum Parsimony), and BI (Bayesian inference) inference analysis resulted in a very similar branching pattern. GenBank accession number, source and name of isolates are shown in Additional file 1: Appendix 1. Two subtypes, PTTV1a-VA and PTTV2b-VA, are proposed subtypes for TTSuV1 and TTSuV2.

One of the phylogenetic trees was constructed using the neighbor-jointing method (Figure 3). In all consensus trees, strains identified in the same genogroup in accordance with sequence synapomorphies were deposited together, and discrepancies among inference algorithms were not reported. Thus, two monophyletic clades were resolved for each inference method, corresponding to genogroups 1 and 2. Phylogenetic studies revealed that there were distinct patterns of clustering for these isolates; however, the phylogenetic tree demonstrated different clusters indicating different subgenogroups infecting pigs from Sichuan (Figure 3). According to the phylogenetic tree, both TTSuV1 and TTSuV2 were made of two subtypes, TTSuV1a, TTSuV1b, TTSuV2a and TTSuV2b. All TTSuV1 strains isolated from Sichuan formed a single clade when compared with other TTSuV1s. TTSuV2-AB439, TTSuV2-DZ, TTSuV2-BZ and TTSuV2-CD strains belonged to a single cluster.

## Discussion

In this study, detailed descriptions of TTSuV infection and circulation in domestic pigs are presented for each subordinate prefecture and city of Sichuan Province, the biggest pig production area in China. Results indicated a high prevalence of TTSuV in domestic pig herds in Sichuan. Prevalence of TTSuV1 was lower than that observed in other eastern and northeastern cities, but higher compared with TTSuV2 prevalence [23]. Of the 244 samples, our results indicate that virus from either genogroup was detected in 203 (83.2%), while 44 animals (18.0%) were co-infected with viruses of both genogroups, and genogroup 2 (76.2%) was more prevalent than genogroup 1 (25%), a situation similar to that reported for many other countries [47]. By statistical analysis, the results indicated that there was statistically significant difference between the prevalence of genogroups 1 infection alone (9.4%, 23/244) and 2 alone (64.8%, 158/244), and between the prevalence of genogroups 2 (76.2%, 186/244) and both genogroups co-infection (18.0%, 44/244) (Figure 1B). Moreover, TTSuV2 (186/244, 76.2%) was more prevalent than TTSuV1 (61/244, 25%). In China, Zhu et al. reported that the frequency of TTSuV1 and TTSuV2 was 8.57 and 6.03%, respectively, however, no pigs were observed to be infected with both TTSuV1 and TTSuV2 as determined by stool analysis [48]. Wu et al. believed infection ratios for both TTSuVs were very high, and no significant difference was observed between TTSuV1 and TTSuV2 [37]. TTSuV prevalence could be the result of surroundings, the sanitary status of farms, and variations in the genus *Iotatorquevirus*[37]. A high TTSuV-positive rate was 78.9% (146/185) in pig clinical samples collected during 2008–2009 from 11 different regions(Anhui, Beijing, Guangxi, Henan, Hunan, Jiangsu, Jiangxi, Shan-dong, Shanghai, Xinjiang and Zhejiang), and there were co-infection with multiple TTSuV strains in the same pig [27]. And a total of three tissue samples (3/20, 15%) from post-weaning multisystemic wasting syndrome-affected pigs and 30 blood samples (30/40, 75%) from healthy pigs were positive for Torque teno sus virus 1 (TTSuV1) and/or 2 (TTSuV2) [26]. By comparison, we could find the prevalent of TTSuVs was much higher in Sichuan than the eastern coastal cities. Overall, the number of TTSuV1 infection was less among all the prefectures and cities investigated. All animals were negative TTSuV1 in seven of 21 prefectures and cities. TTSuV2 infection is more frequent in intensively managed farm-like herds as well as small herds. In spite of wide limited number of samples, our results suggest that some discrepancy exists in the prevalence of TTSuV throughout Sichuan Province. To better understand the prevalence of TTSuV in China, more samples need to be collected and analyzed.

The current study did not investigate the potential pathogenicity of TTSuV infection in pig. Sera were collected from different pig herds, with unknown health condition. The role of TTSuV interaction with other pathogens remains to be elucidated. Persistent human TTV infection could cause an increase in the levels of alanine amino transferase and TTV viremia, with a potential link to many diseases [49]. The mechanism(s) of infection and pathogenicity of TTV and TTSuV remain poorly understood. Due to its ubiquitous presence worldwide, and the lack of morphological or molecular abnormalities for TTV- and TTSuV-infected cells, studying the biological functions of TTV and TTSuV is particularly challenging [39,50,51]. Our results suggested that TTSuV has adapted to its various hosts and is circulating with similar epidemiology. Our study indicated that the clustering of TTSuV isolates might not be associated with geographic origins.

To date, two TTSuVs, TTSuV1 and TTSuV2, have been described in pig herds [6]. The nucleotide sequence variation between both TTSuVs depended greatly on the region of the viral genome analyzed [10,22]. The complete genome length was distinct, even among species in the same genogroup. In this study, the sequencing results showed that TTSuV nucleotide sequence identity in different pig breeds ranged from 82.3–100 and 72.2–100% for genogroups 1 and 2, respectively.

Evaluation of the UTR among TTSuV genogroups as a reliable molecular marker to detect divergence within TTSuV genogroups returned unambiguous results. Saturation and selection pressure in the studied DNA fragment was lacking, and the genetic diversity was great enough to cause discrepancy within TTSuV genogroups. Our analyses suggested that the UTR could be a molecular marker of TTSuV for detection and surveillance. The results from our study support the finding of Segalés [12,47]. Four visible clusters were evident in the phylogenetic tree, which could be generally classified into four subtypes. Although the TTSuV UTR is a good molecular marker, their sequence represents less than 10% of the total genome (2.8 kb). However, other regions of the TTSuV genome could be under higher mutation pressure, such as ORF1 (which encodes the capsid and replication-associated protein with the largest size relative to the other three predicted viral proteins) [52], therefore more useful for evaluating phylogenetic evolution. This has been suggested for PCV2 [53,54], a virus similar to TTSuV. Further investigation of the ORFs, partial or complete would be desirable in future phylogenetic studies [12].

## Conclusions

In conclusion, this study indicates that TTSuV genogroup 1 or 2 infection in pigs varies depending on geographic regions, and that TTSuV infection is ubiquitous across Sichuan Province. Additionally, the TTSuV UTR was determined to be a useful molecular marker in phylogenetic analysis for TTSuV detection and surveillance. Our conclusions are preliminary and can be changed if more appropriated genetic data were obtained. And both genogroups 1 and 2 could be further divided into two subtypes. TTSuV1 subtype b and the two TTSuV2 subtypes are prevalent in Sichuan Province. Our findings lay the basis for further studies aimed at the rapid diagnosis of TTSuV infection and determining the role of this virus in Chinese pig herds. The identification of novel TTSuV strains from pigs in China also paves the way for future disease characterization and genotyping of TTSuV.

## Methods

### Origin of pig sera

A total of 244 pig serum samples were used in the study, taken during the samplings for the monitoring of diseases and antibody response in pigs at various ages, from 23 farms of 21 distinct geographic regions across Sichuan province from 2010 to 2011. We have got written permission from each farmer, National Animal Experiment Teaching Demonstration Center of Sichuan Agricultural University and Animal Disease Prevention and Control Center in Sichuan Province. The general health condition of herds was not known. The animals from which specimens were collected, was handled in accordance with animal protection law of the People’s Republic of China (a draft of an animal protection law in China released on September 18, 2009). This study was approved by the National Institute of Animal Health, Animal Care and Use Committee at Sichuan Agricultural University (approval number 2009–012).

### Extraction of viral DNA from serum

All serum samples were stored at −70°C until DNA extraction. Each serum sample (1 ml) was clarified by centrifugation and subjected to three freeze-thaw cycles. Viral DNA was extracted with phenol-chloroform, precipitated with ethanol, and collected after centrifugation, then resuspended in 50 μl of TE (10 mM Tris–HCl pH 8.0, 1 mM EDTA).

### Nested polymerase chain reaction (nPCR)

Presence of TTSuV genogroups 1 and/or 2 in pig samples was determined using a previously published nPCR method [10]. All primers used in the study were synthesized by Invitrogen (Shanghai, China). The amplicons (272 bp for TTSuV1, 225 bp for TTSuV2) from 10 μl nPCRs were visualized using electrophoresis on 1% (w/v) agarose gels stained with ethidium bromide (10 mg/ml), and analyzed under blue light using a gel imaging system (Bio-Rad, USA). Two or three randomly selected nPCR amplicons (TTSuV1 and TTSuV2), corresponding to every intensive commercial pig farm in Sichuan, were excised from the agarose gels and purified using an Agarose Gel DNA Purification Kit (Biomed Co., Beijing, China), and stored at −20°C.

### Microbial strain and vector

*Escherichia coli* DH5α competent cells used in this study were obtained from the Key Laboratory of Animal Biotechnology Center of Sichuan Province, College of Veterinary Medicine of Sichuan Agricultural University. The pMD 19-T Simple vector was purchased from TaKaRa (Dalian, China). A Plasmid Mini Kit was purchased from OMEGA (Bingjing, China).

### TA cloning, sequencing

Purified amplicons were cloned into pMD19 T Simple in a 10 μl ligation reaction (6 h, 16°C). The ligation reaction comprised 0.5 μl of pMD19 T Simple, 4.5 μl of amplicons, and 5.0 μl of ligation solution. Recombinant plasmids, pMD19 T-V1 and pMD19 T-V2, were transformed into competent *E. coli* DH5α cells, then isolated using a Plasmid Mini Kit (OMEGA, Beijing, China). Gene sequences were sequenced by Invitrogen (Shanghai, China), edited using DNAStar, and aligned with ClustalW. The nucleotide sequences obtained in this study (5 for TTSuV1, 27 for TTSuV2) were deposited into GenBank (Accession numbers JF906766–JF906783, and JN559360–JN559371).

### Phylogenetic analysis of porcine TTSuV sequences

Phylogenetic analysis of the TTSuV UTR was conducted using the method described by Segalés [12]. First, loss of phylogenetic information caused by substitution saturation and several measures of diversity (nucleotide diversity, mean number of mutations per sequence and mean distance within and between genogroups) were assessed. The level of saturation was investigated by plotting the pairwise number of observed transitions and transversions versus genetic distance. Additionally, substitution saturation was evaluated with Xia’s test [55]. All these analyses were performed via the DAMBE program [56]. Previously published methods [57] were then used to calculate whether DNA sequences evolved under a neutral model of selection using DnaSP (version 5.10) [58]. To avoid loss of power and accuracy in phylogenetic estimations [59], a DNA substitution model that best fitted the available data was explored using jModelTest [60].

### Phylogenetic studies

Nucleotide sequences of 25 other *Anelloviridae* family members were obtained from GenBank. Detailed information regarding the sequences of these analyzed viruses is presented in Additional file 1. Multiple alignment of these sequences was performed using MEGA 5 (version 5.0) with ClustalW. In order to avoid introducing a desired mutations into a gene, so alignments containing overlapping (125 bp) genogroup sequences that were removed primers were used to construct a phylogenetic tree [11]. These alignments contained overlapping genogroup 1 sequences of about 225 bp, and genogroup 2 sequences that overlapped by 184 bp, to calculate phylogenetic distances using DNAStar. Phylogenetic and molecular evolutionary analyses were performed via the neighbor-joining method with 1000 bootstrap replicates in MEGA 5. Meanwhile, he inference methods of Maximum Likelihood (ML), Maximum Parsimony (MP) and Bayesian Inference (BI) were also used to evaluate the accuracy of phylogenetic tree analyzed by N-J method. Statistical significance of the branching was estimated using SEQBOOT (1000 resamplings) to build a consensus tree [10].

### Statistical analyses

Statistical analyses were performed with Prism software (GraphPad).The unpaired or paired Student’s *t* test was used to compare the average prevalence of both TTSuV genogroups in general, as well as between single-infected (one or other TTSuV genogroup) and co-infected (by both TTV genogroup) animals. The level of significance was set to P values of <0.05.

## Abbreviations

TTSuV: Torque teno sus virus; nPCR: nested polymerase chain reaction; ssDNA: single-stranded deoxyribonucleic acid; ORF: Open reading frame; UTR: Un-translated region; PMWS: Post-weaning multisystemic wasting syndrome; PCV2: Porcine circovirus type 2; qPCR: Quantitative polymerase chain reaction; PRRSV: Porcine reproductive and respiratory syndrome virus; AIC: Akaike information criterion.

## Competing interests

The authors have no conflicts of interest to declare.

## Authors’ contributions

MM, LZ and ZWX designed the experiments. LZ and MM carried out the sequencing reactions, processed and assembled the sequence reads, and compared the consensus sequences. MM and YFW performed the assays. YCZ, HCW and SL downloaded the reference sequences from GenBank. WZG and ZWX provided intellectual, physical, and financial support for these experiments. All authors read and approved the final manuscript.

## Supplementary Material

Additional file 1: Appendix 1 Detailed information regarding Anellovirus strains used in the analysis. Click here for file
