# Supplementary material for: Molecular investigation of Torque teno sus virus in geographically distinct porcine breeding herds of Sichuan, China
Source: Virol J. 2013 May 24;10:161. doi: 10.1186/1743-422X-10-161 (PMC3679838; doi:10.1186/1743-422X-10-161)
Supplement: Additional file 1: Appendix 1 — Detailed information regarding Anellovirus strains used in the analysis. [file 1743-422X-10-161-S1.docx]

Appendix 1. Detailed information regarding Anellovirus strains used in the analysis.

| Species of porcine TTV genogroups | Strain | Host or isolation source | Country of isolation^a^ | Accession number | Sequences spans (base pairs) | Submited/ Collection yaear^b^ |
| --- | --- | --- | --- | --- | --- | --- |
| Torque teno sus virus 1 | TTV1-ABWC | Porcine | Shichuan, China | JN559361.1 | 272 | 2011/2010 |
| Torque teno sus virus 1 | TTV1-GZ1735 | Porcine | Shichuan, China | JN559360.1 | 272 | 2011/2010 |
| Torque teno sus virus 1 | TTV1-MS | Porcine | Shichuan, China | JF906769.1 | 271 | 2011/2010 |
| Torque teno sus virus 1 | TTV1-YA | Porcine | Shichuan, China | JF906768.1 | 272 | 2011/2010 |
| Torque teno sus virus 1 | TTV1-SN | Porcine | Shichuan, China | JF906766.1 | 272 | 2011/2010 |
| Torque teno sus virus 1 | TTV1-SC1 | Porcine | Shichuan, China | JF694116.1 | 2852 | 2011/2010 |
| Torque teno sus virus 1 | TTV1-SC2 | Porcine | Shichuan, China | JF694117.1 | 2817 | 2011/2010 |
| Torque teno sus virus 1 | TTV-SH0822/2008 | Porcine | Shanghai,China | GU450331 | 2823 | 2010/2008 |
| Torque teno sus virus 1 | TTV1-G26 | Porcine | Spain | GU570202 | 2910 | 2010/- |
| Torque teno sus virus 1 | TTV1-G21 | Porcine | Spain | GU570201 | 2910 | 2010/- |
| Torque teno sus virus 1 | TTV1-1914 | Porcine | Spain | GU570200 | 2913 | 2010/- |
| Torque teno sus virus 1 | TTV1-20N | Porcine | Spain | GU570199 | 2913 | 2010/- |
| Torque teno sus virus 1 | TTV1-19N | Porcine | Spain | GU570198 | 2913 | 2010/- |
| Torque teno sus virus 1 | swSTHY-TT27 | Porcine | Canada | GQ120664 | 2875 | 2009/2005 |
| Torque teno sus virus 1 | 1p | Porcine | Brazil | AY823990 | 2872 | 2004/- |
| Torque teno sus virus 1 | PTTV1b-VA | Porcine | USA | GU456384 | 2875 | 2010/2008 |
| Torque teno sus virus 1 | PTTV1aVA | Porcine | USA | GU456383 | 2878 | 2010/2008 |
| Torque teno sus virus 1 | TTV1-471819 | Porcine | Germany | GU188045 | 2863 | 2009/2008 |
| Torque teno sus virus 1 | TTV1Ln23-1 | Porcine | Liaoning, China | HM633258 | 2875 | 2010/2009 |
| Torque teno sus virus 1 | TTV1Hlj5 | Porcine | Heilongjiang, China | HM633254 | 2878 | 2010/2009 |
| Torque teno sus virus 1 | TTV1Gx3-1 | Porcine | Guangxi, China | HM633253 | 2868 | 2010/2009 |
| Torque teno sus virus 1 | TTV1Fj3 | Porcine | Fujian, China | HM633252 | 2897 | 2010/2009 |
| Torque teno sus virus 1 | TTV1Bj10 | Porcine | Beijing, China | HM633251 | 2914 | 2010/2009 |
| Torque teno sus virus 2 | TTV2-PZH2 | Porcine | Shichuan, China | JN559371.1 | 226 | 2011/2010 |
| Torque teno sus virus 2 | TTV2-PZH1 | Porcine | Shichuan, China | JN559370.1 | 226 | 2011/2010 |
| Torque teno sus virus 2 | TTV2-CDJT23 | Porcine | Shichuan, China | JN559369.1 | 226 | 2011/2010 |
| Torque teno sus virus 2 | TTV2-LZ31 | Porcine | Shichuan, China | JN559368.1 | 226 | 2011/2010 |
| Torque teno sus virus 2 | TTV2-DYZJ15 | Porcine | Shichuan, China | JN559367.1 | 226 | 2011/2010 |
| Torque teno sus virus 2 | TTV2-DYZJ11 | Porcine | Shichuan, China | JN559366.1 | 226 | 2011/2010 |
| Torque teno sus virus 2 | TTV2-MY6 | Porcine | Shichuan, China | JN559365.1 | 226 | 2011/2010 |
| Torque teno sus virus 2 | TTV2-GY8 | Porcine | Shichuan, China | JN559364.1 | 226 | 2011/2010 |
| Torque teno sus virus 2 | TTV2-DZXH1 | Porcine | Shichuan, China | JN559363.1 | 226 | 2011/2010 |
| Torque teno sus virus 2 | TTV2-AB439 | Porcine | Shichuan, China | JN559362.1 | 224 | 2011/2010 |
| Torque teno sus virus 2 | TTV2-LS | Porcine | Shichuan, China | JF906783.1 | 226 | 2011/2010 |
| Torque teno sus virus 2 | TTV2-YB | Porcine | Shichuan, China | JF906782.1 | 226 | 2011/2010 |
| Torque teno sus virus 2 | TTV2-ZG | Porcine | Shichuan, China | JF906781.1 | 226 | 2011/2010 |
| Torque teno sus virus 2 | TTV2-YA | Porcine | Shichuan, China | JF906780.1 | 226 | 2011/2010 |
| Torque teno sus virus 2 | TTV2-BZ | Porcine | Shichuan, China | JF906779.1 | 226 | 2011/2010 |
| Torque teno sus virus 2 | TTV2-NC | Porcine | Shichuan, China | JF906778.1 | 226 | 2011/2010 |
| Torque teno sus virus 2 | TTV2-NJ | Porcine | Shichuan, China | JF906777.1 | 226 | 2011/2010 |
| Torque teno sus virus 2 | TTV2-MS | Porcine | Shichuan, China | JF906776.1 | 226 | 2011/2010 |
| Torque teno sus virus 2 | TTV2-AB | Porcine | Shichuan, China | JF906775.1 | 226 | 2011/2010 |
| Torque teno sus virus 2 | TTV2-GZ | Porcine | Shichuan, China | JF906774.1 | 225 | 2011/2010 |
| Torque teno sus virus 2 | TTV2-CD | Porcine | Shichuan, China | JF906773.1 | 227 | 2011/2010 |
| Torque teno sus virus 2 | TTV2-ZY | Porcine | Shichuan, China | JF906772.1 | 226 | 2011/2010 |
| Torque teno sus virus 2 | TTV2-GA | Porcine | Shichuan, China | JF906771.1 | 226 | 2011/2010 |
| Torque teno sus virus 2 | TTV2-DZ | Porcine | Shichuan, China | JF906770.1 | 225 | 2011/2010 |
| Torque teno sus virus 2 | TTV2-SN | Porcine | Shichuan, China | JF906767.1 | 226 | 2011/2010 |
| Torque teno sus virus 2 | TTV2-SC2 | Porcine | China | JF694118.1 | 2798 | 2011/2010 |
| Torque teno sus virus 2 | TTV2-SC | Porcine | China | HQ204188.1 | 2802 | 2011/2010 |
| Torque teno sus virus 2 | TTV2_1907 | Porcine | Spain | GU570203 | 2744 | 2010/- |
| Torque teno sus virus 2 | 2p | Porcine | Brazil | AY823991.1 | 2735 | 2004/- |
| Torque teno sus virus 2 | PTTV2b-VA | Porcine | USA | GU456385 | 2750 | 2010/2008 |
| Torque teno sus virus 2 | TTV2-472142 | Porcine | Germany | GU188046 | 2802 | 2009/2008 |
| Torque teno sus virus 2 | TTV2Ln13 | Porcine | Liaoning, China | HM633235 | 2822 | 2010/2009 |
| Torque teno sus virus 2 | TTV2Jx1 | Porcine | Jiangxi,China | HM633233 | 2807 | 2010/2009 |
| Torque teno sus virus 2 | TTV2Gx1 | Porcine | Guangxi,China | HM633229 | 2834 | 2010/2009 |
| Torque teno sus virus 2 | TTV2Bj8 | Porcine | Beijing,China | HM633226 | 2824 | 2010/2009 |
| Torque teno sus virus 2 | TTV2Hb1 | Porcine | Hebei,China | HM633225 | 2805 | 2010/2009 |
| Torque teno sus virus 2 | TTV2Jl2 | Porcine | Jilin,China | HM633222 | 2805 | 2010/2009 |
| Torque teno virus 26 | Mf-TTV3 | Japanese macaque | Japan | AB041958.2 | 3798 | 2000/- |
| Torque teno canis virus | Cf-TTV10 | Dog | Japan | AB076002.1 | 2797 | 2001/- |
| California sea_lion_anellovirus | California sea_lion_anellovirus | Zalophus californianus | USA | FJ459582 | 2140 | 2008/- |

^a^ Chinese isolates were able to be traced back to Provinces.

^b^ “-” indicates that information was not available.
